# Influence of Peer-Based Needle Exchange Programs on Mental Health Status in People Who Inject Drugs: A Nationwide New Zealand Study

**DOI:** 10.3389/fpsyt.2016.00211

**Published:** 2017-01-18

**Authors:** Bianca Hay, Charles Henderson, John Maltby, Juan J. Canales

**Affiliations:** ^1^Department of Psychology, University of Canterbury, Christchurch, New Zealand; ^2^New Zealand Needle Exchange Programme, Christchurch, New Zealand; ^3^Department of Neuroscience, Psychology and Behaviour, University of Leicester, Leicester, UK

**Keywords:** needle exchange, peer support, mental health, depression, anxiety, drug safety

## Abstract

Alleviating the personal and social burden associated with substance use disorders requires the implementation of a comprehensive strategy, including outreach, education, community interventions, psychiatric treatment, and access to needle exchange programs (NEP), where peer support may be available. Given that substantial research underscores the potential benefits of peer support in psychiatric interventions, we aimed to conduct a national survey to examine key domains of mental health status in people who inject drugs (PWID) in New Zealand. PWID were recruited from 24 pharmacies and 16 dedicated peer-based needle exchanges (PBNEs) across the country. We focused on two mental health outcomes: (1) affective dysregulation, across the three emotional domains of the Depression Anxiety Stress Scale, due to its role in the maintenance of continued drug use, and (2) positive cognition and effective health- and drug-related information exchange with the provider, using the Satisfaction with Life Scale and an *ad hoc* questionnaire, respectively, in view of their association with improved mental health outcomes. We hypothesized that access to peer support would be associated with mental health benefits for PWIDs. Remarkably, the results of a multistep regression analysis revealed that irrespective of sex, age, ethnicity, main drug used, length of drug use, and frequency of visits to the NEP, the exclusive or preferential use of PBNEs predicted significantly lower depression and anxiety scores, greater satisfaction with life, and increased health-related information exchange with the service provider. These findings demonstrate for the first time an association between access to peer support at PBNEs and positive indices of mental health, lending strong support to the effective integration of such peer-delivered NEP services into the network of mental health services for PWID worldwide.

## Introduction

It is estimated that approximately 16 million people worldwide inject drugs (1). Sharing of drug taking equipment continues to be a major cause of transmission of blood-borne viruses, such as HIV and hepatitis C. Such negative association is especially burdensome in countries with no or poor roll-out needle exchange programs (NEP) and is accentuated by a lack of awareness and education about safe injecting (2). Albeit the services provided vary significantly from country to country, the implementation of harm reduction strategies, currently defined as a comprehensive package aimed at the prevention, treatment, and care for people who inject drugs (PWID), is widely recognized as a satisfactory evidence-based approach to minimize the health risks associated with injecting drugs (3). Key elements of such intervention are the provision of safe equipment and materials, provision of up-to-date information about safe injecting practices, and access to health and counseling referrals.

Stimulated by changes of drug policy in the Netherlands that shifted the focus from detoxification to harm reduction, an environment permissive to self-organization of PWID led to the formation of the first underground exchanges (the “junkiebonden”) in the early 1980s. Some years later, fueled by the positive impact these organizations were having on the community, funding initiatives from the Dutch Ministry of Health led to a spread of NEP schemes throughout the country (4). In New Zealand, the number of notified cases of AIDS had been growing through the 1980s (5), and in 1987, the government decriminalized the sale of needle and syringes, implementing in 1988 the first nationwide NEP in the world (6, 7). Initially conceived as a pharmacy and general practitioner service, the New Zealand scheme quickly expanded with the addition of drug user groups contracted to provide educational support, later constituting charitable trusts and independent NEP operating a peer-to-peer system. At present, there are some 180 pharmacy outlets and 21 peer-based needle exchanges (PBNEs) participating in the New Zealand NEP scheme (www.needle.co.nz).

The peer-based strategy, which in New Zealand has evolved from the peer user groups of the 1980s, is an organic process whereby clients, rather than staff, provide direct service to their peers, particularly in the distribution of needles, syringes, and associated injection equipment. Building on existing community networks, trained peers assist PWID to access exchangers, distribute information about safer drug use and safer sex, and facilitate referrals to other user-friendly health services. Although not exempt of limitations and shortcomings (8), the peer service model, in addition to disseminating safer practices and making a variety of health services more accessible, emphasizes the importance of reconstructing relationships with family, friends, and the community at large (9, 10), which may ultimately contribute to promote greater psychological stability and enhance the benefits of psychiatric interventions. In the area of mental health, there is considerable theoretical and experiential support for the notion that peer support may exert a positive influence in multiple relevant domains, including empowerment (11), symptom distress (12), self-esteem (13), and social integration (14). However, no previous studies have investigated the potential psychiatric benefits of peer support in the context of NEP on a large scale. We conducted a nationwide survey aimed at exploring the psychological well-being of PWID using pharmacies and PBNEs across New Zealand.

In the present study, we drew on the well-established triad of affect, cognition, and behavior, as key psychological mechanisms influencing health outcomes (15). By using this framework, we assessed two psychological health outcomes: (i) affect and (ii) cognitions and behaviors. First, we examined measures of general affect prompted by the close association between positive and negative affect states as possible inductors or outcomes for sustained drug use (16). Second, we assessed the extent to which individuals demonstrated positive cognitions about life and felt able to both share personal health-related information and access information on safe drug use, as these cognitions and behaviors are linked to wellness, enhanced risk perception, and self-efficacy (17).

## Materials and Methods

### Sample

Peer-based needle exchanges and pharmacies (Levels 1 and 2 outlets, see www.needle.co.nz for a complete description of services and type) under the NEP scheme received the questionnaires by post. Included with the questionnaires there was a prepaid postal bag and an instructions sheet for staff. At each pharmacy or peer-based outlet, there was one main contact person (usually the manager of the pharmacy or NEP) who was briefed personally or over the phone and instructed to inform all staff to hand out the survey to PWID. PWID were asked to participate in the survey and those who expressed willingness were first given an information sheet with details of the general purpose of the study. The information sheet indicated that the information provided by participants would remain anonymous at all times and that through completion of the survey consent would be obtained to use the data for analysis and publication. For this purpose, each NEP was asked to allocate a private space within their premises. No interviewer was present to prevent bias. The survey was completed by the PWID with no time limit. The estimated time to complete it was 30 min approximately. To compensate for the time invested in completing the questionnaire, a NZ $10 supermarket voucher was given to the participants. Surveys were collected from 315 respondents (170 males, 141 females, with 4 who did not disclose their sex), aged from 19 to 66 years (mean age = 42.40, SD = 9.2), from regions throughout the north (Auckland, Palmerston, Midlands, Wellington, Northland, and Napier) and south (Christchurch/Canterbury, Dunedin/Southland, and Nelson/Malborough) islands of New Zealand, including 24 pharmacies (16 in the north island and 8 in the south island) and 16 PBNEs (11 in the north island and 5 in the south island). The study was approved by the Human Ethics Committee of the University of Canterbury and by the New Zealand NEP, the Pharmaceutical Society, and the Pharmacy Guild of New Zealand. The authors assert that all procedures contributing to this work comply with the ethical standards of the relevant national and institutional committees on human experimentation and with the Helsinki Declaration of 1975, as revised in 2008. Table 1 provides a summary of other demographic and drug use data relating to ethnicity, type and main drug used, length of drug use, preferred use of NEP, and frequency of visits to the NEP.

**Table 1:** **Demographic and drug use data relating to ethnicity, occupation, type and main drug used, length of drug use, preferred use of NEP, and frequency of visits to the NEP**.

Demographic/drug use variable	Frequency
Ethnicity	New Zealand European (*n* = 233)
	Maori (*n* = 65)
	Pasifika (*n* = 6)
	Other (*n* = 5)
	Asian (*n* = 3)
	Missing (*n* = 3)

Occupation	Unemployed/benefits (*n* = 149)
	General laborer and service (*n* = 47)
	Homemaker (*n* = 36)
	Semi-professional/professional (*n* = 29)
	Sales and marketing (*n* = 19)
	Semi-skilled laborer or service (*n* = 12), student/volunteer, pension (*n* = 7)
	Arts (*n* = 3)
	Self-employed (*n* = 2)
	Missing (*n* = 11)

Main drug type	Opiates (*n* = 188)
	Stimulants (*n* = 91)
	Other (*n* = 20)
	Missing (*n* = 16)

Type of drugs taken in the last month	Methadone (*n* = 183)
	Morphine (*n* = 166)
	Amphetamines (*n* = 126)
	Ritalin (*n* = 103)
	Benzodiazepines (*n* = 32)
	Homebake (*n* = 26)
	Other (*n* = 14)
	Cocaine (*n* = 8)
	Opium (*n* = 7)
	Anabolic (*n* = 4)
	Ecstasy (*n* = 3)
	Cyclizine (*n* = 2)
	Benzylpiperazine (*n* = 2)
	Tramadol (*n* = 1)
	Methylone (*n* = 1)
	Palfium (*n* = 1)

Length of use	More then 10+ years (*n* = 218)
	8–9 years (*n* = 18)
	6–7 years (*n* = 18)
	4–5 years (*n* = 21)
	2–3 years (*n* = 24)
	0–1 year (*n* = 10)
	Missing (*n* = 6)

Preferential use of the peer-based needle exchange	I always use peer-based needle exchange (*n* = 154)
	Not always, but nearly every time I use the peer-based needle exchange (*n* = 24)
	I tend to use the peer-based needle exchange more often (*n* = 10)
	I use the peer-based needle exchange and the pharmacies about the same number of times (*n* = 20)
	I tend to use the pharmacies more often (*n* = 13)
	Not always, but nearly every time I use the pharmacies (*n* = 14)
	I always use the pharmacies (*n* = 76)
	Missing (*n* = 4)

Frequency of visits to NEP	1–2 times a month (*n* = 118)
	3–4 times a month (*n* = 96)
	5–6 times a month (*n* = 38)
	7+ times a month (*n* = 58)
	Missing (*n* = 5)

### Assessment of Affective State

To assess the psychological status of the PWID in the affective domain, the Depression Anxiety Stress Scale 21 (DASS-21), a short form of Lovibond and Lovibond’s 42-item self-report, was utilized (18). DASS-21 comprises three dimensions of seven items each: depression, anxiety, and stress. The scale aims to capture an individual’s distress by evaluating these three common negative emotional states. Each question is answered on a 4-point Likert scale: 0 (never), 1 (sometimes), 2 (often), and 3 (almost always). Evidence has shown the reliability and validity (i.e., internal consistency and test-retest) of the scale in both clinical and non-clinical settings (18–20).

### Assessment of Well-Being

The Diener Satisfaction with Life Scale (SWLS) was used as a reliable indicator of subjective well-being (21). This scale consists of five items designed to measure global cognitive judgments of one’s life satisfaction. The participant is required to answer each question on a 7-point Likert scale: 1 (strongly disagree) to 7 (strongly agree). Past research supports the reliability and validity of this scale (22).

### Assessment of Health-Related Information Exchange

To examine the self-perceived ability of PWID to share experiential information in relation to their drug use, including health information, and to access information on drug use and safe practices, a 5-point Likert questionnaire consisting of eight *ad hoc* questions was devised. This health-related information exchange questionnaire (IEQ) included questions aimed to measure the degree to which PWID were willing to share personal experiences at the NEP, felt they had an adequate knowledge about safe practices, felt comfortable about asking questions regarding drug use and health referral services, and perceived the information available at the NEP as being useful and effective.

## Results

### Missing Data

Across the 10 variables for which data were collected [of which three of the perceived variables (negative affect, satisfaction with life and information exchange) comprised 32 items], 90 cases were removed from the analysis due to missing data, leaving a cohort size of *n* = 225 (124 males, 101 females, mean age = 42.37, SD = 9.12). A series of independent group *t* tests and chi-square test [we used SPSS for Windows 22 (IBM Corp, 2013) for all statistical tests] were computed for those variables for whom individuals were removed due to having some missing data [ranging from *n* = 67 to 86 (as data remained for some of these individuals for other variables in the analysis)] and those individuals included in the analysis (*n* = 225). Table 2 shows that no statistical significant differences occurred for mean scores between both groups for the established variables: DASS, SWLS, age, length of use, frequency of visits to the NEP, and preferred use of NEP. Furthermore, no significant association was found between inclusion and exclusion due to missing data for sex (χ^2^ = 0.07, *p* = 0.797), ethnicity (χ^2^ = 0.09, *p* = 0.765), and main drug of use (χ^2^ = 3.03, *p* = 0.082).

**Table 2:** **Mean scores for health-related information exchange, Depression Anxiety Stress Scale (DASS) subscales, satisfaction with life, preferred use of needle exchange programs (NEP), frequency of visits, and age by people who inject drugs included and excluded from the analysis**.

Variable	Excluded (*n*)	Mean	SD	Included (*n*)	Mean	SD	*t*	*p*
Health-related information exchange	75	31.52	6.21	225	31.96	6.13	0.58	0.592
Depression (DASS)	74	16.54	12.74	225	15.68	10.50	0.45	0.652
Anxiety (DASS)	67	14.15	12.26	225	13.72	9.88	0.30	0.766
Stress (DASS)	71	17.80	12.54	225	17.13	10.44	0.45	0.652
Satisfaction with life	85	17.87	8.24	225	16.80	7.25	1.12	0.265
Use of NEP	86	3.62	2.65	225	3.03	2.52	1.81	0.072
Length of use	84	5.00	1.60	225	5.20	1.46	−1.07	0.286
Frequency of visits	85	2.00	1.04	225	2.16	1.14	−1.13	0.260
Age	74	42.50	9.70	225	42.39	9.12	0.11	0.916

### Clinical Caseness

To demonstrate the clinical casesness of affective state among the respondents, the DASS provides severity ratings for each of the subscales of depression, anxiety, and stress. Table 3 shows a breakdown of the scores by each of the severity rating provided by the DASS manual scoring.

**Table 3:** **Clinical caseness of depression, anxiety, and stress scores for the current sample as determined by severity rating provided in the Depression Anxiety Stress Scale manual**.

Severity	Depression		Anxiety		Stress	
	Frequency	%	Frequency	%	Frequency	%
Normal	71	31.6	70	31.1	98	43.6
Mild	32	14.2	20	8.9	38	16.9
Moderate	50	22.2	46	20.4	31	13.8
Severe	34	15.1	24	10.7	39	17.3
Extremely severe	38	16.9	65	28.9	19	8.4

### Factor Analysis

With the measures we collected, we identified three possible domains: emotional evaluations of affect (comprising depression, anxiety, and stress of the DASS), cognitive evaluations of affect (comprising SWLS scores), and health-related information exchange (comprising the IEQ scores). To explore these as three separate outcome domains, we performed an exploratory factor analysis to explore the underlying structure of the items from the DASS, SWLS, and IEQ. The number of participants (225) to variables (34) ratio (6.6:1) exceeded the recommended minimum ratio needed for EFA of 5 to 1 (with a minimum number of participants of 150) (23, 24). All items were subjected to maximum likelihood analysis (Kaiser–Meyer–Olkin measure of sampling adequacy = 0.92; Bartlett’s test of sphericity, (^2^ = 4,749.93, *df* = 561, *p* < 0.001). To determine the number of factors to extract, we performed a parallel analysis of Monte Carlo simulations (25) that allowed us to determine the number of factors by comparing the eigenvalues of a higher value with those that might be expected from purely random data. The fourth eigenvalue (11.40, 4.79, 2.62, and 1.26) failed to exceed the fourth mean eigenvalue (1.82, 1.71, 1.63, and 1.56) calculated from 1,000 generated data sets with 225 cases and 34 variables, suggesting that a 3-factor solution was appropriate. Therefore, a three-factor solution was explored using a promax rotation, as we expected the factors to be correlated, with delta set to 0. The results of this analysis are presented in Table 4.

**Table 4:** **Exploratory factor analysis of the DASS, satisfaction with life, and health-related information exchange items**.

Items	Factor		
	1	2	3
I found it hard to wind down	0.577	–0.038	0.062
I was aware of dryness of my mouth	0.326	–0.064	0.118
I couldn’t seem to experience any positive feeling at all	0.478	–0.042	–0.096
I experienced breathing difficulty (e.g., excessively rapid breathing, breathlessness in the absence of physical exertion)	0.516	–0.077	0.069
I found it difficult to work up the initiative to do things	0.512	–0.097	–0.094
I tended to overreact to situations	0.729	0.022	–0.006
I experienced trembling (e.g., in the hands)	0.612	–0.032	0.097
I felt that I was using a lot of nervous energy	0.824	0.020	0.079
I was worried about situations in which I might panic and make a fool of myself	0.856	0.050	0.198
I felt that I had nothing to look forward to	0.632	0.001	–0.202
I found myself getting agitated	0.773	–0.022	0.001
I found it difficult to relax	0.755	0.048	–0.022
I felt down-hearted and blue	0.659	–0.074	–0.171
I was intolerant of anything that kept me from getting on with what I was doing	0.717	–0.012	0.042
I felt I was close to panic	0.813	0.021	0.061
I was unable to become enthusiastic about anything	0.672	–0.013	–0.141
I felt I wasn’t worth much as a person	0.652	0.034	–0.244
I felt that I was rather touchy	0.727	0.072	–0.047
I was aware of the action of my heart in the absence of physical exertion (e.g., sense of heart rate increase, heart missing a beat)	0.734	–0.030	0.171
I felt scared without any good reason	0.825	0.023	0.072
I felt that life was meaningless	0.644	0.015	–0.237
In most ways my life is close to my ideal	0.048	0.041	0.819
The conditions of my life are excellent	0.131	–0.027	0.909
I am satisfied with my life	–0.043	–0.042	0.797
So far I have gotten the important things I want in life	–0.044	–0.051	0.654
If I could live my life over, I would change almost nothing	0.090	0.020	0.542
Do you feel your needle exchange provides an environment where you feel safe?	–0.032	0.704	–0.045
Do you feel you know enough regarding injecting safely?	–0.140	0.559	–0.101
Do you feel comfortable to ask your needle exchange questions on drug use?	–0.042	0.734	–0.036
Do you share personal experiences regarding your drug use with the needle exchange?	0.003	0.522	–0.024
Does the needle exchange provide information on safe drug use?	0.074	0.878	0.107
Do you feel well informed on safe drug use?	–0.061	0.815	0.019
Do you feel your needle exchange is willing to answer questions you may have or are willing to refer you to other support services?	0.071	0.892	0.035
Does the needle exchange offer information regarding the accessibility of user-friendly disposal of used needles?	–0.031	0.737	–0.014

Meaningful loadings were assessed using the criteria of 0.32 (“poor”), 0.45 (“fair”), 0.55 (“good”), 0.63 (“very good”), and 0.71 (“excellent”) (26). From this solution, all the items from the DASS loaded on the first factor, with one item loading with a value of 0.33 and the rest of loadings ranging from 0.48 to 0.86. All the IEQ items loaded on the second factor with one item loading with a value of 0.34, and the rest of the loading values ranging from 0.52 to 0.89. Finally, all the SWLS items loaded on the third factor, with loading values ranging from 0.54 to 0.91. Moreover, the correlations between the factors ranged from −0.10 to −0.50, suggesting the factors had no more than 25% of shared variance. Therefore, the current findings suggest that the current items used fall under three different general domain: emotional evaluations of affect (in which higher scores represent higher levels of negative emotional affect), cognitive evaluations of affect (in which higher scores represent higher levels of positive cognitive affect), and self-perceived ability to share and access relevant health information (in which higher scores represent higher levels of health-related information exchange).

However, the finding that the items of the DASS load on one factor in this context questions whether the DASS scores is best represented, at least in this sample, by one overall score, or is best considered in terms of three factors, given theoretical and empirical evidence that the DASS commonly forms three factors of depression, anxiety, and stress (18). Therefore, we subjected just the items of the DASS scale to a confirmatory factor analysis (CFA). A key focus of CFA is to demonstrate the incremental value of proposed models (27). We compared the goodness of fit, a unidimensional model, representing an underlying latent factor structure of negative affect with a three-factor model comprising depression, anxiety, and stress. To assess the goodness of fit of the data, we looked at the five statistics recommended by Hu and Bentner (28) and Kline (29): the relative χ^2^ (CMIN/DF), the comparative fit index (CFI), the non-normed fit index (NNFI), the root mean square error of approximation (RMSEA), and the standardized root mean square residual (SRMR). The following are the criteria to assess whether the model fit was “acceptable”: (i) CMIN/DF should be less than three to be acceptable, (ii) the CFI, GFI, and NNFI should exceed 0.90 to be acceptable, (iii) the RMSEA should not exceed 0.08 and be below 0.06 to be a “good” fit, and (iv) the SRMR values less than 0.08 are “acceptable” and those less than 0.05 are “good” (28, 29). Improvement on the value of the model was assessed by changes in CFI (ΔCFI) being > 0.01 (30).

Comparing the two models, the three-factor model (CMIN/DF = 2.02, CFI = 0.93, GFI = 0.87, NNFI = 0.92, RMSEA = 0.068, SRMR = 0.046) shows acceptable and improved (as indicated by ΔCFI being > 0.01) goodness-of-fit statistics over the one-factor model (CMIN/DF = 2.98, CFI = 0.87, GFI = 0.78, NNFI = 0.85, RMSEA = 0.094, SRMR = 0.059). Therefore, we computed the following scale scores to represent three domains, in terms of overall scores for SWLS (α = 0.85) to assess cognitive evaluation of affect, overall scores for IEQ to assess information sharing (α = 0.87), and the three DASS subscale scores for depression (α = 0.90), anxiety (α = 0.84), and stress (α = 0.90) to assess emotional evaluations of affect.

### Multiple Regression

We ran 5 three-step multiple regressions, with overall SWLS and IEQ scores and DASS subscales scores used in the series as dependent variables, with sex, age, and ethnicity (recoded for NZ European [1] versus non-NZ European [2]) as the predictor variables in step 1; then main drug used (recoded as opiate [1] versus stimulant [2]), length of use, and frequency of visits to the NEP as predictor variables in step 2; and, finally, we incorporated preferred use of NEP as a predictor variable in step 3. An *a priori* sample size calculator for a multiple regression study, given a desired probability level of *p* < 0.05, the number of predictors in the model being 7, the anticipated effect size being medium (*f^2^* = 0.15), and the desired statistical power level of .8, calculated the minimum required sample size as *n* = 103, suggesting that our current sample size of *n* = 227 used for this analysis exceeded these criteria. The variance inflation factors (VIFs) and tolerance factors for each of the single predictor variables were no larger than 1.20 and no smaller than 0.83, respectively. Therefore, they did not contravene the threshold values for VIF of at least 5 and tolerance statistics of less than 0.2 that are used to suggest collinearity between independent variables (30).

**Figure 1:**
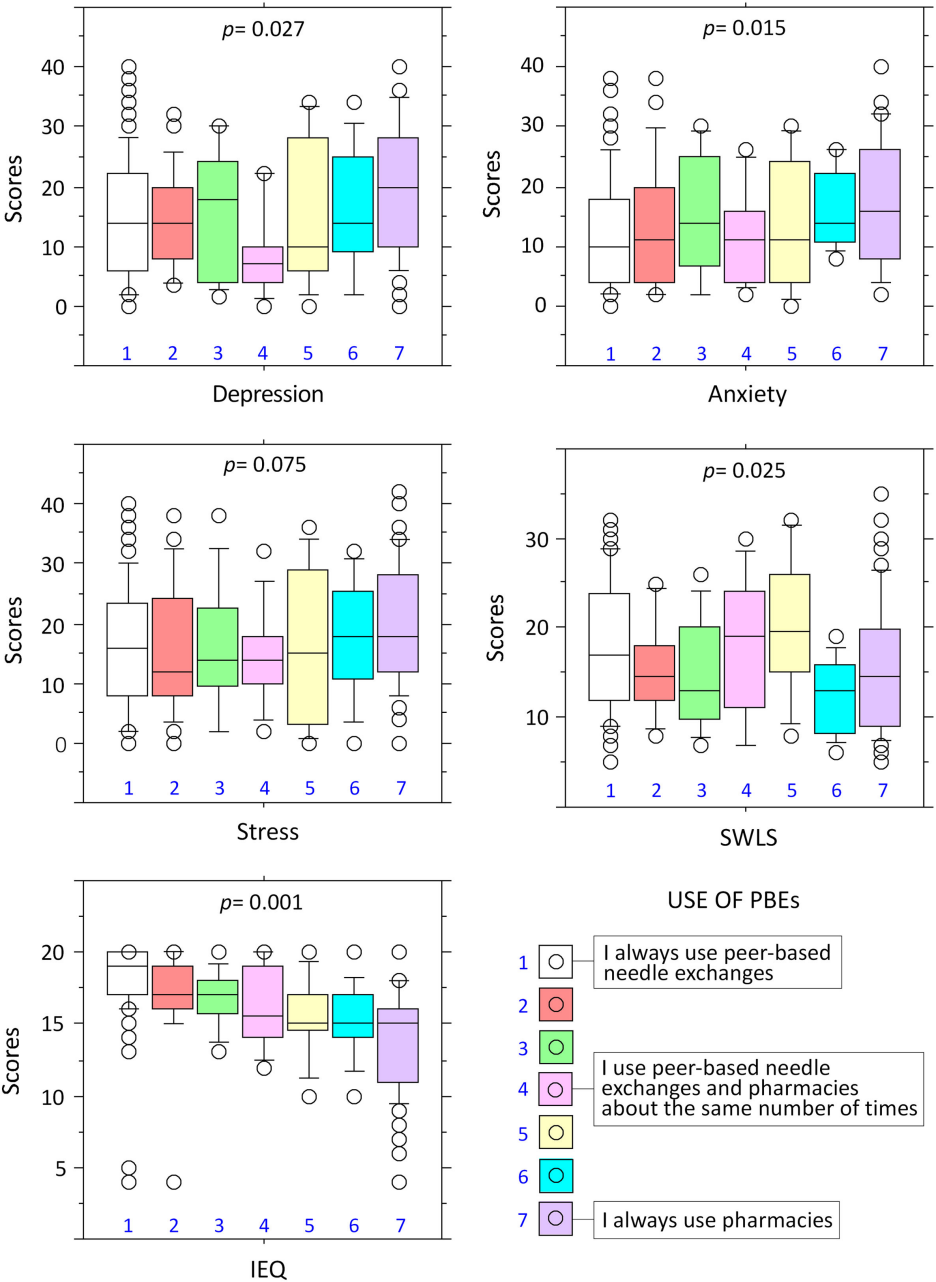
**Exclusive of preferential use of peer-based needle exchanges predicts lower levels of depression and anxiety, heightened satisfaction with life, and increased health- and drug-relevant information exchange**. Box plots show the different levels of the predictor variable, measured with a 7-point Likert scale, and the scores obtained in the different domains of the Depression Anxiety Stress Scale (i.e., depression, anxiety, and stress), the satisfaction with life (SWLS) and the IEQ. The 7-point Likert scale included the following options for the use of peer-based needle exchanges or pharmacies: (1) I always use peer-based needle exchange, (2) not always, but nearly every time I use the peer-based needle exchange, (3) I tend to use the peer-based needle exchange more often, (4) I use the peer-based needle exchange and the pharmacies about the same number of times, (5) I tend to use the pharmacies more often, (6) not always, but nearly every time I use the pharmacies, and (7) I always use the pharmacies. The *p* values indicate the statistical significance of the correlations between preferential use of needle exchanges with measures of depression, anxiety, stress, SWLS, and information exchange questionnaire (IEQ).

For each regression (see Table 5) in step 1 (depression, *F*[3,221] = 0.25, *r* = 0.06, *r*^2^ = 0.01, adj *r*^2^ = 0.01, *p* = 0.861; anxiety, *F*[3,221] = 0.42, *r* = 0.08, *r*^2^ = 0.01, adj *r*^2^ = 0.01, *p* = 0.741; stress, *F*[3,221] = 1.66, *r* = 0.15, *r*^2^ = 0.02, adj *r*^2^ = 0.01, *p* = 0.175; satisfaction with life, *F*[3,221] = 1.22, *r* = 0.13, *r*^2^ = 0.02, adj *r*^2^ = 0.01, *p* = 0.305; health-related information exchange, *F* [3,221] = 0.68, *r* = 0.10, *r*^2^ = 0.01, adj *r*^2^ = 0.01, *p* = 0.818) and step 2 (depression, Δ*R*^2^ = 0.01, *p* = 0.852; anxiety, Δ*R*^2^ = 0.01, *p* = 0.857, stress, Δ*R*^2^ = 0.01, *p* = 0.958; satisfaction with life, Δ*R*^2^ = 0.01, *p* = 0.795; health-related information exchange, Δ*R*^2^ = 0.02, *p* = 0.174), the predictor variables failed demonstrate statistical significance in predicting each dimension. In step 3, the inclusion of the preference site for the NEP led to a statistically significant change in *R*^2^ for depression (Δ*R*^2^ = 0.02, *p* = 0.027), anxiety (Δ*R*^2^ = 0.03, *p* = 0.015), satisfaction with life (Δ*R*^2^ = 0.02, *p* = 0.025), and health-related information exchange (Δ*R*^2^ = 0.32, *p* < 0.001), but not stress, although the effect was marginally significant (Δ*R*^2^ = 0.02, *p* = 0.075). Data points relating to preferred use of NEP and each of the dependent variables are shown in Figure 1.

**Table 5:** **Multiple regression analysis with, Depression Anxiety Stress Scale subscales, satisfaction with life and health-related information exchange scores used as a dependent variable, with sex, age, and ethnicity as the predictor variables in step 1; then main drug used, length of use, and frequency of visits to NEP as predictor variables in step 2; and, finally, preferred use of NEP as a predictor variable in step 3**.

	Depression				Anxiety				Stress			
	*B*	β	*t*	*p*	*B*	β	*t*	*p*	*B*	β	*t*	*p*
**Step 1**												
Sex	–0.01	–0.01	–0.01	0.991	0.39	0.04	0.57	0.568	–0.48	–0.05	–0.68	0.499
Age	–0.03	–0.05	–0.77	0.442	–0.01	–0.03	–0.38	0.706	–0.06	–0.11	–1.58	0.115
Ethnicity	0.18	0.03	0.37	0.712	0.38	0.06	0.82	0.415	0.73	0.10	1.51	0.132
**Step 2**												
Main drug	–0.01	–0.01	–0.01	0.991	0.59	0.06	0.77	0.441	–0.06	–0.01	–0.08	0.940
Length of drug use	–0.03	–0.05	–0.77	0.442	0.04	0.01	0.14	0.889	–0.06	–0.02	–0.22	0.823
Frequency of visit to NEP	0.18	0.03	0.37	0.712	–0.12	–0.03	–0.41	0.683	0.16	0.04	0.53	0.599
**Step 3**												
Preference for use of NEP	0.31	0.15	2.22	0.027	0.32	0.16	2.45	0.015	0.25	0.12	1.79	0.075

	**Satisfaction with life**				**Health-related information exchange**							
	***B***	**β**	***t***	***p***	***B***	**β**	***t***	***p***				

**Step 1**												
Sex	0.06	0.03	0.43	0.669	0.10	0.05	0.77	0.443				
Age	0.01	0.11	1.64	0.103	0.01	0.03	0.45	0.657				
Ethnicity	0.09	0.07	0.99	0.325	0.10	0.08	1.15	0.250				
**Step 2**												
Main drug	–0.10	–0.05	–0.72	0.474	–0.25	–0.12	–1.67	0.096				
Length of drug use	0.02	0.03	0.45	0.654	–0.08	–0.12	–1.70	0.091				
Frequency of visit to NEP	–0.03	–0.04	–0.52	0.606	0.01	0.01	0.22	0.830				
**Step 3**												
Preference for use of NEP	–0.06	–0.15	–2.25	0.025	–0.22	–0.57	–10.44	0.001				

*B = unstandardized beta coefficient; β = standardized beta coefficient; t = t value; p = probability; NEP = needle exchange point (lower scores represent preferences for peer-based exchanges)*.

## Discussion

Although community development programs providing access to injecting equipment exist in other countries, including European countries, Australia, and USA, there is a considerable resistance from governments to support integrated NEP of the kind currently implemented in New Zealand (31, 32), with some NEP in Europe having been recently dismantled due to political opposition (33). In many countries, there remains considerable counterproductive, not evidence based, sociopolitical debate over the ethical and financial implications of harm reduction strategies, despite its proven cost effectiveness (3, 34). Such debate fails to address not only the consequences for public health in terms of transmission of blood-borne viruses but also the psychological and social needs of vulnerable and marginalized populations. NEP activities are enabled in New Zealand by the Ministry of Health through dedicated (peer based) exchanges, mobile units, and participating pharmacy outlets. Pharmacies operate at different levels depending on the type of injecting equipment provided and distribute educational materials to PWID on safe injecting. Dedicated exchanges and associated mobile units are run and staffed largely by paid workers who have had life experiences as injecting drug users themselves and therefore understand the lifestyle and challenges PWID face. Albeit training is sparse, such workers are well placed to offer advice on safe injecting and available referral health services as appropriate. The emphasis of these PBNEs is not only on harm reduction but also on a model of intentional psychosocial support and outreach, where the peers employed in the support role are considered to be further along in their recovery journey. Therefore, they are capable and willing to provide leadership, advice, shared life experiences, and emotional support to those who enter their services (35, 36). Sharing credible positive experiences, practical strategies, and coping mechanisms has long been regarded as a form of support that people with experience in dealing successfully with personal mental health problems can offer others facing similar challenges (12, 37). Albeit the harm reduction strategy implemented by way of NEP is widely recognized to minimize the health risks associated with injecting drugs (1), scientific evidence that peer support can be of psychological benefit for PWID in this specific context is lacking.

The current study was the first to examine on large-scale key aspects linked to mental health status and welfare of PWID using peer-based and non-peer-based NEP schemes. Participants were recruited from 24 pharmacies and 16 PBNEs across the country of New Zealand. We focused on three aspects of well-being, including emotional evaluations of negative emotional symptomatology (specifically depression, anxiety and stress) (38), cognitive evaluations of affect (satisfaction with life), and health-related information exchange, as both predictors and outcomes that might be linked to improved mental health status and recovery in PWID (16). First, the results indicated that the sample of PWID comprised a large number of subjects with psychiatric vulnerability, as revealed by the severity rating of the DASS (Table 3). Second, the findings clearly showed that the preference for PBNEs over pharmacy-based services (exchanges) shared unique variance with lower depression, lower anxiety, greater satisfaction with life, and increased health-related information exchange, while controlling for sex, age, ethnicity, main drug used, length of drug use, and frequency of visits to the NEP. In terms of the salience of these findings, considering the size of unstandardized and standardized beta coefficient, that effect was larger for the health-related information exchange, with comparatively smaller associations for depression, anxiety, and satisfaction with life. Notwithstanding, these data show for the first time that the use of PBNEs, in comparison with other similar services, is associated with positive mental health outcomes.

Where peers are employed as providers of services and support within traditional or specialized health agencies, peer support is generally framed within a model of wellness focused on effective functioning and recovery rather than the illness and its specific symptoms (8). A core feature defining the effectiveness of this model is the ability of the peer to engage with clients on the same level through increased empathy and deeper understanding of their challenges (39). Previous evidence suggests that such community-oriented, peer-delivered interactions have the potential to engage hard to reach or marginalized groups and positively impact on multiple layers of risk and behavioral change and particularly on people who regularly use drugs (40–42). Peer support has been consistently linked in the mental health literature with enhanced acceptance, self-esteem, self-efficacy, quality of life, community inclusion, empowerment, and willingness to work toward recovery through exposure to role modeling and alternative, more functional, worldviews (8, 12, 13, 43). The current study was correlational in nature and therefore did not allow us to identify the specific mechanisms mediating the positive effects of peer services on the well-being and affective state of PWID. Notwithstanding, the findings for positive mental health outcomes and effective health-related information exchange variables revealed an association of the peer support service with positive enhancement processes, which may occur *via* the self or social factors (11, 14). Given the nature of the data and the fact that observations were collected concurrently, threefold interactions may have given rise to such enhancement processes. First, both higher levels of effective health-related information exchange and reduced affective dysregulation could lead to a preference for PBNEs *via* heightened perceived well-being and self-efficacy, through a process akin to self-empowerment (11). Second, a preference for PBNEs might reflect elevated levels of social activation (14), which in turn could promote greater levels of positive mental health and more fluid health-related information exchange with the service provider. The third possibility is that the two processes combine together, be it within individuals, or across the sample.

In terms of recommendations for public and mental health policies, the current findings have far-reaching implications for the development of effective programs of harm reduction and especially for the psychological support and, in cases of psychiatric co-morbidity, rehabilitation of PWID. We showed here that patent psychological benefits were associated with access to peer support at PBNEs in the areas of well-being, affective regulation, and effective communication with the service providers. Such benefits are likely to impact positively at multiple levels, both psychological and social, and could go a long way in facilitating remission from continued drug use, recovery, and strengthening of social networks within the community. Taken together, these findings highlight the need to standardize peer support roles in terms of their values, skills, knowledge base, and remit and for further integration of such peer-supported programs within national policies regulating NEP and health care for PWID worldwide.

## Ethics Statement

The study was approved by the Human Ethics Committee of the University of Canterbury and by the New Zealand NEP, the Pharmaceutical Society, and the Pharmacy Guild of New Zealand. PWID were asked to participate in the survey by staff at PBNEs and pharmacies and those who expressed willingness were first given an information sheet with details of the general purpose of the study. The information sheet indicated that the information provided by participants would remain anonymous at all times and that through completion of the survey consent would be obtained to use the data for analysis and publication. No additional considerations.

## Author Contributions

BH, CH, and JC designed the study; BH collected the data; JM and JC analyzed the data; and JM and JC wrote the paper.

## Conflict of Interest Statement

The authors declare that the research was conducted in the absence of any commercial or financial relationships that could be construed as a potential conflict of interest.
